# p38 MAPK and PI3K/AKT Signalling Cascades inParkinson’s Disease

**Published:** 2015

**Authors:** Saurabh Kumar Jha, Niraj Kumar Jha, Rohan Kar, Rashmi K Ambasta, Pravir Kumar

**Affiliations:** 1 *Molecular Neuroscience and Functional Genomics Laboratory, Department of Biotechnology, Delhi Technological University (Formerly DCE), Delhi, India.*; 2 *Department of Neurology, Tufts University School of Medicine, Boston, MA (USA).*

**Keywords:** Parkinson’s disease (PD), p38MAPK, PI3K/AKT, neuroinflammation, oxidative stress (OS), apoptosis, neurotherapeutics

## Abstract

Parkinson's disease (PD) is a chronic neurodegenerative condition which has the second largest incidence rate among all other neurodegenerative disorders barring Alzheimer's disease (AD). Currently there is no cure and researchers continue to probe the therapeutic prospect in cell cultures and animal models of PD. Out of the several factors contributing to PD prognosis, the role of p38 MAPK (Mitogen activated protein-kinase) and PI3K/AKT signalling module in PD brains is crucial because the impaired balance between the pro- apoptotic and anti-apoptotic pathways trigger unwanted phenotypes such as microglia activation, neuroinflammation, oxidative stress and apoptosis. These factors continue challenging the brain homeostasis in initial stages thereby essentially assisting the dopaminergic (DA) neurons towards progressive degeneration in PD. Neurotherapeutics against PD shall then be targeted against the misregulated accomplices of the p38 and PI3K/AKT cascades. In this review, we have outlined many such established mechanisms involving the p38 MAPK and PI3K/AKT pathways which can offer therapeutic windows for the rectification of aberrant DA neuronal dynamics in PD brains.

Neurodegenerative disorders (NDs) continue to traumatize an aging proportion of the human population especially in the industrialized world. Aging has long been recognized as a compound process of damage accretion that ultimately leads to noticeable disruption of multiple cellular and molecular proceedings, which ultimately are translated into various chronic ailments such as Alzheimer's disease (AD), multiple sclerosis (MS), Parkinson's disease (PD), atherosclerosis and many more (1-3)**.** Although, several NDs have a pharmacological treatment, which as in the case of AD, PD, epilepsy and MS slow down the course of the disease, and are restricted to damage limitation, but are not equipped enough to annul the effects or for that reason heal the infirm. Sadly though, the future of such ambitious modalities currently hangs on morbid conjecture and fragile hopes and thus the current focus of the research bevy is to primarily delve unprecedented mechanisms that shall in future restrain the cardinal effects in NDs and also presumably act as custodians of permanent cure (4, 5).

PD is a chronic, neurodegenerative state and the second most commonly observed brain disorder (the most common being AD) which impacts nearly 1% of the global population aged 65 and older. Incidentally, PD appears to be less prevalent among Asian population as compared to the Western world and it is unclear whether this is in a way allied to the extensive use of traditional medicine in the Eastern half of the planet (6). Nevertheless, the use of complementary and alternative medicine (CAM) has been reported to be as high as 76% in countries like Korea. PD is typically characterized by the progressive loss of muscle control, impaired balance, slowness, akinesia, bradykinesia, tremors, postural instability, and decline in striatal dopamine levels of the central nervous system (CNS), and rigidity observed due to the significant loss of dopaminergic (DA) neurons in the substantia nigra (SN) in the midbrain (7). Interestingly, only 10% of all PD cases are caused by genetic mutations, and animal models previously used to comprehend these mutations revealed a significant insight into the loss-of-function status of α-synuclein and LRRK2 particularly in autosomal dominant PD and PINK1/Parkin and DJ-1 in autosomal recessive cases. These findings remain crucial since they represent possible therapeutic targets, however, in the face of such advances, the precise etiology of PD still remains uncertain. Multiple lines of evidence from molecular and cellular to epidemiological studies suggest that innate and environmental factors such as aging, genetics, 1-methy l- 4- pheny l -1, 2, 3, 6- tetrahydropyridine (MPTP), 6-hydroxydopamine (6-OHDA) metals, mitochondrial dysfunction induced by environmental toxins, such as mitochondrial complex I inhibitors rotenone, traumatic brain injury, and shortage of trophic factors that can play a role in PD neuropathology (8-10). In addition, lifestyle factors viz. cigarette smoking and coffee consumption, with a gender bias can also influence the onset of PD. Notably; the neurotoxin 6-hydroxydopamine (6-OHDA) is extensively used to mimic the PD associated neurodegeneration in both *in vivo *and *in vitro *experimental models. On the treatment front, the use of levodopa, dopamine agonists, herbal medicines, health supplement foods, and acupuncture are on the rise all over the world among patients afflicted with PD, however, without much benefit (9, 11, 12).

In the last 15 years, the procedure of deep brain stimulation (DBS) has emerged as touchstone to mitigate the adverse neuropathlogical symptoms witnessed in advanced forms of the disease. The procedure is mainly designated for PD victims who are dopamine-responsive but with disabling motor complications such as motor fluctuation, dyskinesia, or unendurable side-effects of anti-PD suppositories. It is well known that motor fluctuations like wearing-off and peak-dose dyskinesia are motor anomalies observed few years after patients are started on medical treatment. When these complications breach the edge of severity, despite maximal fine-tuning of pharmacological agents, DBS has been shown to be effective and safe with benefits lasting for no less than 10 years. In fact, bilateral sub thalamic nucleus DBS has arisen as a treatment of choice and proven to have an unquestionable influence on motor symptoms, countenancing the minimization of drug treatment and its side effects. Numerous other reports have demonstrated the effectiveness of neurosurgery, specifically on motor symptoms and on health related quality of life. However, a vital concern has been that most of those studies have testified no significant perfection in social adaptation after subthalamic nucleus deep brain stimulation in some patients. Furthermore, the lack of post-operative improvement in the psychosocial dimension of health-related quality of life and its link to coping strategies is still unclear and there are contentious reports surrounding the use of DBS in the early stage of PD and most medication centers will offer this modality typically to infirm with disruptive motor complications obstinate to drug treatment (13, 14). Therefore, it is of urgency to examine novel mechanisms that can be used as a benchmark therapy of choice in patients bloated with Parkinson’s in future.

It is well documented that oxidative stress (OS), impaired ROS/NO balance, microglia activation, and chronic inflammation are striking pathological features observed in PD brains. These factors altogether have detrimental effects on the integrity of dopaminergic (DA) neurons, thus, may potentially lead to neuronal apoptosis and subsequent neurodegeneration. Not surprisingly, these critical neuro determinants are under the control of an array of proteins and signalling networks, and accruing genetic studies in recent years have advocated prominently on the role of improper phosphorylation events, dysfunctional kinases, and aberrantly functioning kinases associated signalling events as few of many responsible determinants involved in a convoluted network defining PD progression and pathogenesis. One such mechanism contributing to microglia response and neuroinflammation in both inherited and sporadic forms involve the protein named LRRK2 (leucine-rich repeat kinase 2). LRRK2 can effectively moderate the neuroinflammatory ambience in traumatized neurons in response to a panel of pathological stimuli. LRRK2 is a large multi-domain protein belonging to the cohort of mammalian ROCO (Ras of complex) proteins. It can be functionally characterized by the presence of an enzymatic core, comprising of ROC/GTPase, COR (C-terminus of ROC) and serine threonine kinase domains. In addition, there are multiple protein-protein interaction domains including ankyrin and leucine-rich repeat motifs at the N-terminus, and WD40 repeats at the C-terminus. LRRK2 mutations can induce microglias through hyperpolymerization and hyperphosphorylation of cytoskeleton and vesicle components, thereby, directing these cells towards a pro-inflammatory ambience, which in turn can result in aggravated inflammation and subsequent neurodegeneration. Profound investigation into the innumerable functionalities of misregulated signalling cascades involving kinases such as p38 mitogen-activated protein (MAP) kinase, protein kinase B (AKT) kinase, and C-Jun N-terminal kinase (JNK), extracellular signal-regulated kinases (ERK), PI3K/AKT shall unravel novel mechanism for drug targeting in future. In that regard, the identification of microglia-specific kinase substrates, GTPase downstream effectors, and interactors shall reveal acute therapeutic hot spots and outline credible prototypes for the attenuation of the cardinal symptoms and motor complications in this group of disorders (8, 15, 16).

Numerous intracellular signalling cascades that congregate on MAPK exist in all eukaryotic cells and play critical roles in various cellular activities. The p38 MAPK as also can be designated as stress-activated protein kinase (SAPK), is especially triggered by a range of cytotoxic stress stimuli and cytokines. In response, p38 potentially drive crucial cellular activities such as proliferation, differentiation, survival, and stress-induced apoptosis. In the central nervous system (CNS), p38 MAPK is central towards the maintenance of synaptic plasticity, and as a result, anomalies’ resulting from the deviant functioning of p38 MAPK pathway in neurons has been observed in brain diseases like AD, PD and amyotrophic lateral sclerosis (ALS). Consistent activation of JNK or p38 MAPK is critical towards facilitating neuronal apoptosis in AD, PD and ALS brains (17-19).

On contrary, PI3K/AKT pathway modulates cellular activities like neuronal cell proliferation, migration and plasticity. The cytoprotective phenotype of PI3K/AKT provides an important signalling for neuroprotection, however, it prerequisites that the pathway is optimally activated in PD brains; this could possibly antagonize the detrimental effects of the p38 MAPK activation in degenerating DA neurons and thus, can assist in establishing a neuro-protective setting in insulted brains. In general, the activation of ERK or p42/p44 MAP kinase and the PI3K/AKT pathway encourage cell survival (cytoprotective pathways), whereas SAPK's, c-Jun N-terminal kinases (JNK's) and the p38 MAPK, moderate cell mortality (20, 21). Nevertheless, several plausible cyto-dynamics involving P38 MAPK- PI3K/AKT and their subtle contribution towards progressive neurodegenerative remains an area of active curiosity for research in PD. Moreover, active biomolecules targeting the impaired p38-PI3K/AKT balance could significantly contribute to neuroprotection in PD challenged brains.

In this review, we will focus solely on the viable contributions made by the p38 MAPK and PI3K pathways towards maintaining neuronal dynamics in PD brains. At a later stage we have enumerated a group of probable neurotherapeutics molecules, which can offer neuroprotection by mechanisms involving the likely targeting of the cell survival (PI3K/AKT) and death pathways (p38 MAPK), and actually can attenuate or prevent neurodegenerative symptoms associated with PD brains in future.

## MAP Kinase pathway: At a glance

**p38 MAPK**

p38 MAPK (mitogen-activated protein kinase) signalling cascade provides a mechanism for cells to respond to a catalogue of external mitogens (signals) and respond accordingly by mediating a wide range of cellular effects. In fact, the diversity and specificity in cellular responses as depicted by the cascade is facilitated via a simple linear architecture, which comprises of sequentially operating core of three evolutionarily conserved protein kinases namely; MAPK, MAPK kinase (MAPKK), and MAPKK kinase (MAPKKK). MAPKKKs are serine/threonine kinases, which are activated via phosphorylation and/or because of their interaction with a small GTP-binding protein of the Ras/Rho family in response to extracellular stimulus. MAPKKK activation results in phosphorylation and activation of MAPKKs, which consequently stimulate MAPK activity through dual phosphorylation of threonine and tyrosine residues positioned in the activation loop of kinase subdomain VIII (22). The activated MAPKs now phosphorylates target substrates specifically on serine or threonine residues followed by a proline. MAPKKs such as MEK3 and MEK6 are activated by a wide range of MAPKKKs (MEKK1 to 3, MLK2/3, ASK1, Tpl2, TAK1, and TAO1/2), which themselves become activated in response to oxidative stress, UV irradiation, hypoxia, ischemia, and cytokines, including interleukin-1 (IL-1) and tumor necrosis factor alpha (TNF-α) (23).

At present, five different MAP kinases (MAPks) have been characterized and investigated namely; extracellular signal-regulated kinases (ERKs) 1 and 2 (ERK1/2), c-Jun amino-terminal kinases or stress-activated protein kinases (JNKs/SAPKs) 1, 2, and 3, p38 isoforms α, β, γ, and δ, ERKs 3 and 4, and ERK5. The kinase p38α (p38) was initially isolated as a 38-kDa protein which was observed to be rapidly phosphorylated at tyrosine motifs in response to LPS stimulation. Later, p38 was cloned and studied as a molecule capable of binding puridinyl imidazole derivatives; these derivatives inhibit the biosynthesis of inflammatory mediators like interleukin-1 (IL-1) and tumor-necrosis factor (TNF) in LPS activated monocytes. p38 (also known as CSBP, mHOG1, RK, and SAPK2) kinases are more responsive towards stress stimuli such as osmotic shock, ionizing radiation, and cytokine stimulation and four different variants of p38 arising out of alternative splicing are known viz. p38α, p38β, p38γ (ERK6, SAPK3), and p38δ (SAPK4). Among these, p38 and p38β are ubiquitously expressed in tissues, whereas, p38γ and p38δ show variegated expression in a tissue specific manner (24).

Each of the p38 variants comprises of a Thr-Gly-Tyr (TGY) dual phosphorylation motif and sequence comparison performed earlier suggests that each p38 isoform shares approximately 60% identity with other members of the p38 group but only 40-45% with other MAP kinase family members. The activity of p38 is controlled and coordinated *in vitro* by three different kinases: MKK3, MKK4, and MKK6. *In vivo*, MKK3 and MKK6 are necessary for tumor necrosis factor-stimulated p38 MAPK activation whereas, ultraviolet radiation-mediated p38 MAPK activation requires MKK3, MKK4, and MKK6 (25). 

p38 isoforms can also be stimulated by GPCRs and by Rho family GTPases; Rac and Cdc42. It is interesting to mention here that MAPKs catalyse the phosphorylation and activation of several protein kinases, termed MAPK-activated protein kinases (MKs), which represent an additional enzymatic step in the MAPK catalytic signaling cascade. MEK3 and MEK6 do not participate in the activation of ERK1/2 or JNK and display a high degree of specificity for p38. In addition, MEK4 (MKK4/Sek1) JNK kinase show limited MAPKK activity toward p38. MEK6 is capable of activating all the p38 isoforms, whereas, MEK3 is discerning and preferentially phosphorylates the p38α and p38β isoforms. p38 isoforms are activated as a result of MEK3/6-catalyzed phosphorylation of Thr-Gly-Tyr (TGY) motif in the p38 activation loop. The differential specificity in p38 activation results from the formation of functional complexes between MEK3/6 and different p38 isoforms and the selective recognition of the activation loop of p38 isoforms by MEK3/6. The length of the phosphorylated TGY motif and the activation loop is different in other MAPKs namely ERK2 and JNK, which likely contributes to the p38 substrate specificity. P38 substrates include cPLA2, MNK1/2, MK2/3, HuR, Bax, and Tau in the cytoplasm and ATF1/2/6, MEF2, Elk-1, GADD153, Ets1, p53, and MSK1/2 in the nucleus (26).

Emerging proofs advocate a role for the p38 MAPK and MKP-1 in the maintenance and demise of dopaminergic neurons. Mitogen-activated protein kinase phosphatase-1 (MKP-1) is a negative regulator of p38 activity and other MAPKs such as ERK, and c-Jun NH (2) -terminal kinase (JNK). MKP-1 was found to be expressed in DA neurons cultured from E14 rat ventral mesencephalon (VM) and it was reported that DA neurons when transfected to overexpress MKP-1, triggered a substantial increase in neurite length and branching with maximum upsurge observed in primary branches (27). In addition, DA neurons displaying over-expressed MKP-1 patterns are subjected to neuroprotection against the effects of PD inducing neurotoxin 6-OHDA. MKP-1 can also promote the growth and elaboration of dopaminergic neuronal processes suggesting that MKP-1 is actively involved in DA neuronal maintenance and therefore deviant MKP-1 expression is a hallmark of damaged DA neurons in PD (28). Therefore, formulating strategies aimed at augmenting MKP-1 expression to appropriate p38 activity may be advantageous in shielding dopaminergic neurons from PD induced damage (29).

## PI3K/AKT/mTOR pathway

The PI3K- PKB/ Akt pathway is highly conserved, tightly controlled and a multistep signalling cascade. Since its discovery in the 1980s, lipid kinase termed phosphoinositide 3-kinases (PI3Ks) has been proven time and time again to facilitate crucial cellular dynamics viz. survival, proliferation and differentiation. PI3Ks critically operate downstream of receptor tyrosine kinases (RTKs) and G protein coupled receptors (GPCRs) and are responsible for propagating a wide array of signals arising out from numerous growth factors and cytokines into intracellular communications by generating phospholipids, which in turn activate the serine/threonine kinase AKT and several other effector pathways (30). 

PI3Ks can be divided into three classes based on their structural physiognomies and substrate specificity; of these, the most commonly investigated are the class I enzymes that are activated directly by cell surface receptors. Class I PI3Ks can further be segmented into class IAs that are activated by RTKs, GPCRs and oncogenes like the small G protein Ras, and class IBs, that are entirely moderated by GPCRs. Activated receptors can directly trigger class 1A PI3Ks bound via their regulatory subunit or adapter molecules like the insulin receptor substrate (IRS) proteins. Once activated, class I PI3Ks generate the phospholipid PI(3, 4, 5)P_3_ that serves as a secondary messenger, driving multiple effector pathways influencing key cellular processes. PI3K cascade is negatively regulated by the tumour suppressor PTEN (phosphatase and tensin homolog deleted from chromosome 10) and the cellular levels of PI(3,4,5)P_3_ is closely regulated by the antagonizing PTEN levels. In fact, PTEN down regulates PI3K activity via its intrinsic lipid phosphatase activity that diminishes the cellular pool of PIP3 by converting PI(3,4,5)P_3_ back to PI(4,5)P_2_. Therefore, loss of function of PTEN will result in uncontrolled PI3K signalling thereby leading to baffling disorders like cancer and neurodegeneration (31).

AKT is a serine/ threonine kinase which is expressed as three isoforms, AKT1, AKT2 and AKT3, which are encoded by three PKB genes namely α (PKBα), β (PKBβ), and γ (PKBγ). All the three isoforms can be characterized based upon a parallel architecture comprising of an N-terminal PH domain, a central serine/threonine catalytic domain, and a small C-terminal regulatory domain. Initial AKT activation is facilitated by translocation to the plasma membrane mediated by docking of the PH domain to the membranous phospholipid PI(3,4,5)P_3_, resulting a change in AKT confor-mational and subsequently divulging the two critical amino acid residues for phosphorylation. Phosphorylation at both the exposed sites, T308 by PDK1 and S473 by PDK2, are a prerequisite in order for the AKT to achieve full activation status. Once AKT is activated by phosphorylation at T308 and S473, it then facilitates the phosphorylation of targets viz. GSK3 (glycogen synthase kinase 3) and FOXOs (the forkhead family of transcription factors). A variety of PDKs are currently known to operate in the process, including ILK (integrin-linked kinase), PKCbII, DNA-PK (DNA-dependent protein kinase), and ATM (ataxia telangiectasia mutated), while AKT itself has PDK functionality. However, it is strongly believed that mTORC2 (the mTOR/rictor complex) is the chief source of PDK2 activity under most circumstances (32).

mTOR belongs to a group of Ser/Thr protein kinases group of more distantly related enzymes (related to class I,II and III PI3Ks) and occasionally referred to class IV PI3Ks and includes members like ATM, ATR (ataxia telangiectasia and Rad3 related), DNA-PK and SMG-1 (SMG1 homolog, phosphatidylinositol 3-`kinase-related kinase). PI3K/AKT pathway upstream of mTOR, moderates mTOR activity. mTOR is made up of two distinct complexes namely mTORC1 and mTORC2. The mTORC1 subunit is responsible for mTOR catalytic activity and harbours other components such as Raptor (regulatory associated protein of mTOR), PRAS40 (proline- rich AKT substrate 40 kDa) and the protein mLST8/GbL. Likewise, mTORC2 comprises of mTOR, Rictor (rapamycin insensitive companion of mTOR), mSIN1 (mammalian stress- activated protein kinase interacting protein 1) and mLST8/GbL. AKT is capable of activating mTOR by phosphorylating both PRAS40 and TSC2 (tuberous sclerosis complex) thereby offsetting the inhibitory effects on mTORC1. PKB/Akt binds to PIP3 at the plasma membrane in that way allowing PDK1 to access and phosphorylate the exposed T308 site in the “activation loop,” thus leading to the partial activation of the PKB/Akt component. This amendment is however adequate to stimulate mTORC1 by directly phosphorylation and inactivation of PRAS40 and TSC2 as mentioned above (33). Second phosphorylation of Akt at S473 in the carboxy-terminal hydrophobic motif, either by mTOR or by DNA-PK; result in full activation of PKB/Akt. mTOR can also operate as PDK2 and phosphorylate AKT when it is bound to Rictor in the mTORC2 subunit. mTOR is pivotal to cell growth and proliferation as it monitors variables like monitoring nutrient availability, cellular energy levels, oxygen levels, and mitogenic signals. Well-characterized effector targets of mTORC1 includes 4E-BP1 (4E-binding protein), ribosomal protein S6 kinase and S6K1 (p70S6 kinase) which, in turn, phosphorylates the ribosomal protein S6 (S6/RPS6) (34).

mTOR also regulates autophagy, the failure to which leads to deficiency in the elimination of abnormal and toxic protein aggregates which subsequently trigger catastrophic cellular stress, failure and ultimately death. Autophagy is also modulated by starvation, growth factors, and cellular stressors and has long been proven to play a critical role in PD neuropathology. Yet, the cross-talk between PI3K/AKT/mTOR and autophagy is compound and the comprehensive examination of tissue from patients suffering from PD and of animal and cellular models shall provide further valuable insght (35).

## p38 MAPK mediates microglial response and neuroinflammation.

Microglial cells arise from mesodermal /mesenchymal progenitors and are the resident macrophages in the CNS. Once matured, these cells are disseminated into all regions of the CNS, spread through the brain parenchyma, and attain a specific ramified morphological appearance known as "resting microglia". In the normal brain, microglia however have highly motile procedures by which they patrol their territorial domains. Additionally, these cells can communicate with macroglial cells and neurons and with other cells of the immune system, the interaction mediated by a wide range of cell signalling mechanisms. Microglial cells display characteristic receptors as labelled for brain-specific communication such as neurotransmitter receptors and immune cell-specific such as for cytokines. Interestingly, microglial cells are the most vulnerable sensors of brain homeostasis and upon any detection of anomaly such as traces of brain lesions; nervous system dysfunction or external insults (trauma, toxicants), these cells surpass a multi-staged activation prototype to transform from “resting microglia” to “activated microglia”. The activated microglial cells have the enormous capacity to secrete a repertoire of molecules which can either act damaging or advantageous to the neighbouring population. Activated microglial cells migrate to the site of injury where they proliferate further and phagocytize damaged cells and cellular compartments (36, 37).

## Microglial activation and neuroinflammation in PD pathology

**Fig. 1:**
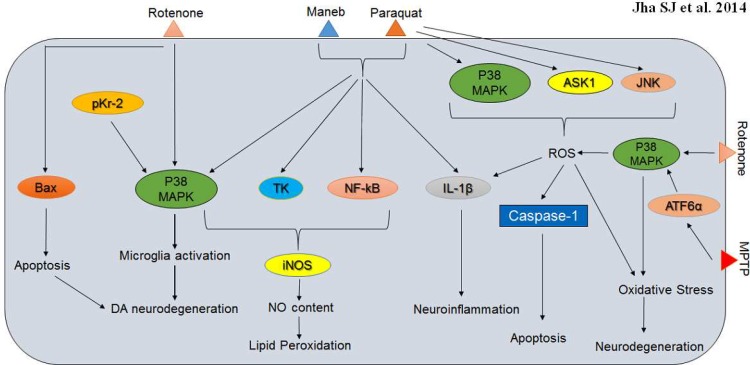
p38 MAPK interactions involved in Parkinson’s disease neuropathology and associated neurodegeneration. Neurotoxins viz. rotenone, maneb, paraquat and MPTP evokes numerous detrimental phenotypes in degenerating neurons and p38 MAPK is responsible for microglia activation, induction of oxidative stress, apoptosis, neuroinflammation and neurodegeneration as triggered by these toxins

Human brain and immune system are convolutedly involved in a crosstalk so as to maintain tissue homeostasis. A panel of evidences obtained from human and animal research have advocated the principle that neuroinflammation significantly contributes to the neuronal loss in PD. Available reports highlight the centrality of non-cell-autonomous pathological mechanisms in PD, which in most cases are regulated by the activation of glial and peripheral immune population. Neuroinflammation in PD is a chronic mechanism that can be connected with the alteration of glial cells, including astrocytes and microglia. Microglia activation in PD brains acutely involves a panel of microglial-derived neurotoxic factors such as reactive oxygen species (ROS), inducible nitric oxide synthase (iNOS), elevated pro-inflammatory cytokine levels, and upregulated inflammatory-associated factors such as cyclooxygenase-2, which altogether cooperate to stabilize microglial response in PD brains (Figure 1). Therefore, and it is not surprising that the prolonged use of anti-inflammatory drugs can indeed reduce the risk for the disease. The neuronal response to microglia activation triggers unwanted trauma viz. oxidative stress (OS), neuroinflammation, cytokine-receptor-mediated apoptosis, which eventually contribute to DA neuronal mortality and subsequent disease progression. Interestingly, recent reports on transgenic mice related model mice were supportive of the idea that neuroinflammation in PD can be ambiguous, that is protective in the initial stages of degeneration but becomes severely damaging as the disease progresses (7, 38-40). 

*In vivo* evidences of neuroinflammation in PD reported the upregulation of inflammatory genes in the periphery and in the CNS, the intrusion of peripheral immune cells into the CNS, and the transformed composition and phenotype of peripheral immune cells. Notably, activated microglial cells are at the heart of the neuro-inflammatory program and the hypothesis has constantly been supported by reports highlighting the neuropathology of PD brains. Initial breakthrough study by McGeer et al.  first reported about the increased microglia status in the substantia nigra *parts compacta* (SNpc) region of post mortem PD brains, following which, several other novel post- mortem studies underlined the significant signs of inflammation and oxidative stress, including increased microglial activation and lipid peroxidation in PD afflicted brains. The midbrain region, which includes the SNpc houses the highest proportion of microglial cells, the resident immune cells of the brain, as compared to the other regions of the brain and this could be decisive towards the heightened sensitivity of this region to inflammatory stimuli. 

Further, microglias respond to a panel of signals which include bacterial and viral products, α-synuclein, complement, antibodies, cytokines, and neuronal death which can occur as a result of response to cytokines such as TNFα, ligation of death receptors such as Fas, and toxicity of reactive oxygen species. Phagocytosis may also trigger microglial activation through loss of inhibitory CD200-CD200R signalling cascade. In response to these wide range of stimuli, microglia further secrete cytokines, ROS, prostanoids that have immune modulatory properties, and chemokines which are responsible for the deployment of peripheral immune cells. Similar to all other cells with antigen-presenting features such as B lymphocytes, macrophages, monocytes, dendritic cells, activated microglia cells also harbors major histocompability class II (MHC-II) molecules on its surface, which are responsible for presenting endocytozed or lysosomal peptides to CD4 T lymphocytes for toxicity clearance. Microglial activation is therefore a major driving force behind dopaminergic neurodegeneration in PD animal models generated using neurotoxins such as rotenone, MPTP and paraquat. Nonetheless, continued microglial activation and neuroinflammation in post-mortem samples and animal models have since been long- established in living PD patients enduring PET scans with the ligand PK11195. Therefore, reducing or preventing the sustained microglial activation might restrict inflammation and may protect the brain from neurodegenerative insults in PD patients (41-44).

## p38 MAPK and associated components together mediate microglial response in PD

It is well-known for quite some time that microglial response is central to DA neuron degeneration and recent studies have proposed that p38 MAPK cascade has a critical impact on microglial activation and response. P38 MAPK together with CD200-CD200R signalling can moderate microglial dynamics in PD brains. CD200-CD200R holds the microglia cells in a quiescent state and PD associated neurodegeneration may well be concomitantly associated with disruption of CD200-CD200R and p38 MAPK signalling axis. This dual signalling axis when operating normally can promote microglia silencing in the SN, which could indeed prevent disease onset and progression. Additionally, p38 MAPK can also activate NADPH oxidase and intercede microglial response. Endogenous molecule(s) like prothrombin kringle-2 (pKr-2) is a domain of human prothrombin distinct from thrombin that has the ability to activate cultured rat brain microglia *in vitro*. Alternatively, prothrombin can trigger NO release and enhance mRNA expression levels of inducible NO synthase, IL-1β, and TNF-α in rat brain microglia. PKr-2 can imitate the effects of prothrombin in promoting NO synthesis and in the upregulation of various inflammatory mediators as mentioned above. Interestingly, both prothrombin and pKr-2 can trigger the same signalling cascades in particular involving p38 MAPK and other kinases such as extracellular signal- regulated kinase½ and c-Jun N-terminal kinase and NF-κB in an analogous manner. Increased NO levels in response to either of these molecules can be diminished by inhibitors such as PD98059 (extracellular signal regulated kinase pathway), SB203580 (p38 MAPK), N-acetylcysteine (NF-κB), Go6976, bisindoly-lmaleimide, and Ro31-8220 (all three against protein kinase C), and D609 and U73122 (both against phospholipase C). Further, pKr-2 can also facilitate the apoptosis of DA neurons in the SN by activating microglial cells via diverse mechanisms involving MAPKs (45-47). Neurotoxins such as maneb and paraquat can similarly activate the microglial cells, escalate the nitrite content, and upgrade the expression levels of IL-1β, p38 MAPK, NF- kB and TK, thereby contributing to DA neurodegeneration. NO can modulate PD pathology by contributing to an increase in maneb and paraquat-induced lipid peroxidation in mouse striatum and molecules such as TK, NF-kB and p38 MAPK are well-acknowledged to modulate iNOS expression. Notably, caffeine can offer neuroprotection by diminishing nitric oxide (NO) production, neuro-inflammation and microglial activation in DA neurons (48, 49). These observations altogether propose that the expression prototype of p38 MAPK and inflammatory mediators are central towards microglial maintenance, inflammation and eventually DA neuron degeneration. 

The environmental toxin rotenone, a mitochondrial complex I inhibitor can directly activate microglial cells through the p38 MAPK pathway and initiate DA neuronal damage in SNpc, which ultimately results in parkinsonism, but the exact mechanisms behind the selective degeneration of nigral DA neurons are not yet fully understood. It was observed that rotenone administration in SNpc of Lewis rats significantly downgraded the motor activity and resulted in loss of THase immunereactivity. Moreover, the degeneration of nigral DA neurons was escorted by an increase in p38 (MAPK), P-p38(MAPK), p53, and Bax expression levels. The neurotoxin exhibited similar effects in cultured PC12 cells and resulted in the upregulation of p38 (MAPK), P-p38(MAPK), p53 and Bax levels, thereby drawing some kind of parallelism between the activation prototypes *in vitro* and *in vivo*. Once activated, Bax is exported into the mitochondrial membrane where it oligomerizes and triggers the mitochondrial apoptotic signalling and the aforesaid observation strongly indicates that p38 (MAPK)/p53-associated stimulation of Bax can indeed contribute to rotenone's neurotoxicity in PD models (50). Lastly, 6-OHDA treatment of MN9D dopaminergic neuronal cells also results in translocation and oligomerization of Bax onto the mitochondria as is the case with the earlier rotenone models. Altogether, these findings suggest that there exist an independent amplification loop of Bax translocation and oligomerization mediated by caspase and p38 MAPK during ROS- mediated DA neurodegeneration (51). An exhaustive investigation is therefore obligatory so as to establish a tangible role of p38 MAPK and auxiliary components in moderating the microglial integrity and activation program and subsequent neurodegeneration in PD brains.

## p38 MAPK mediates oxidative stress in diseased neurons

Neural cells suffer severe functional or sensory loss in neurodegenerative disorders and as discussed in previous sections, microglia- derived inflammatory neurotoxins play a major role in disease pathogenesis. Although, oxygen is the basis for life, defective metabolism and excess ROS production contributes to severe trauma and in contribution with several other environmental or genetic factors, oxidative stress ultimately leads to ROS accumulation in neural cells. Nevertheless, the human body’s innate antioxidant defence plays a decisive role in the prevention of any loss due to ROS but imbalance in defence mechanism of antioxidants, the overproduction or incorporation of free radicals from environment to living system can lead to serious penalty and calamitous neurodegeneration. Moreover, a spectrum of environmental prompts, ER-stress, mitochondrial dysfunction, DNA injury, the accretion of the damaged misfolded proteins due to defective proteasome function, neuroinflammation, tissue damage and subsequent neural cell apoptosis also subject the brain to severe insults. These factors affect glial function thereby aiding to hasten the cadence of the disease. Understanding the connections between oxidative stress, free radical formation, neuro-inflammation, and neurotoxicity is critical to deciphering novel disease mechanisms and the development of model neurotherapeutics to antagonize disease progression (52- 54). Oxidative stress in DA neurons can trigger the p38 MAPK pathway thus leading to the activation of both mitochondrial and extra mitochondrial apoptotic pathways PD culture models. These results suggest that oxidative stress and p38 MAPK pathways operate to balance the pro and anti- apoptotic phenotypes of DA neurons (55).

Paraquat (PQ) elicits a dose-dependent increase in ROS which results in death of SH-SY5Y neuroblastoma cells. This observation can be closely associated with the activation of ASK1 and the stress kinases p38 and JNK SH-SY5Y cells. It has recently reported that the chemical inhibition of either p38 or JNK can confer resistance from L-DOPA-induced apoptosis. Moreover, direct knockdown of ASK1 protects from L-DOPA- induced neuronal cell death. Furthermore, the suppression of the 6-OHDA- generated ROS by treating the cells with N-acetyl-L-cysteine effectively constrains the 6-OHDA- triggered activation of ASK1, p38 and JNK, and thereby protects the cells from apoptosis. It must be noted here that ROS mediated caspase-1 activation and mature IL-1β release are strictly reliant on the p38 MAPK levels in 6-OHDA model systems. These studies clearly show the path from ROS generation to the initiation of p38/JNK signalling via activation of ASK1 and subsequent apoptosis in investigated PD systems (56-57). Rotenone can also meritoriously generate ROS, the concentration levels of which can be directly correlated with the activity of p38 MAPK in the microglia populace (8, 51). These studies clearly show the path from ROS generation to initiation of p38/JNK signalling via the activation of ASK1 and subsequent apoptosis in investigated PD systems.

ATF6α is an ER-membrane-bound transcrip-tion factor in mammalian cells that is activated as a consequence of protein misfolding in the ER. ATF6α functions as a critical regulator of ER quality control. 1-Methyl-4-phenyl-1, 2, 3, 6-tetrahydropyridine (MPTP), a dopaminergic neurotoxin well-known to generate OS, activates ATF6α and increases the level of ER chaperones and ER-associated degradation (ERAD) component in DA neurons. This induced oxidative stress not only stimulates phosphorylation of p38 MAPK but also augments the interaction between phosphorylated p38MAPK and ATF6α, leading to an increment in the transcriptional activity of ATF6α. This mechanism provides a credible link between oxidative stress and ER stress by underscoring the reputation of ATF6α in the protection of the DA neurons from MPTP induced neurotoxicity that occurs via OS-induced activation of ATF6α and p38MAPK- mediated enrichment of ATF6α transcriptional activity (59). Mutations in PINK1 (phosphatase and tensin homolog (PTEN)-induced putative kinase 1) gene is causative behind autosomal recessive PD. Recent studies have investigated the impact of PINK1 on HO-1 (heme oxygenase-1) activation in SH-SY5Y cell lines following H_2_O_2_ or 1-methyl-4-phenylpyridinium [MPP (+)] treatment. It was suggested that the H_2_O_2 _induced HO-1 induction was dependent on Akt and ERK phosphorylation. Moreover, in cells expressing PINK1 G309D mutant and the knockdown of tumour necrosis factor receptor- associated protein-1(TRAP1), the phosphorylation of ERK and Akt was inhibited but not p38 MAPK phosphorylation. These results identified a novel mechanism involving p38 MAPK by which the defect in PINK1 inhibits the oxidative stress- induced HO-1 production. Above all, aberrant HO-1 production following oxidative stress hastens the DA neurodegeneration and directs the brain to a traumatic state in PD patients with PINK1 defect (60). Finally, the uncharacteristic expression of matrix metalloproteinases (MMPs) play their part in PD prognosis and contributing factors such as ROS, PI3K, NF-κB, and AP-1 are commonly involved in 6-OHDA- and MPP (+)- induced MMP-9 gene expression during PD. SK-N-BE(2)C human neuroblastoma and Cath.a mouse DA cell lines when treated with 6-OHDA and MPP(+), resulted in an induction of MMP-9 expression, where the role of p38 MAPK was found to be only differential (61).

## PI3K/AKT/mTOR pathway mediates neuroprot-ection in PD

Accumulating evidences strongly suggest on PI3K/Akt and mTOR to being neuroprotective and hence malfunctioned in PD brains; this is actually of relevance to longevity and may present strategic targets for therapeutic improvement (62). Recent research statistics strongly advise that the vulnerability of DA neuron could arise from elevated metabolic stress levels, resulting from numerous perturbed cascades designated for the control of energy metabolism and cell survival in response to growth factors, oxidative stress, and nutrient deprivation (PI3K/AKT, mTOR, eIF4/p70S6K and Hif-1α). Altogether, these factors operate in a convoluted network thereby adding to archetypal phenotypes observed in PD patients. One of the cardinal symptom observed in diseased brains is neuroinflammation and PTEN induced putative kinase 1 (PINK1), an autosomal recessive familial PD gene, regulates the inflammatory ambience during traumatic states. Dearth in PINK1 levels expedites neuro-inflammation in PD brains through diminished AKT activation and enhanced IκB degradation in response to traumatic brain injury (63). In fact, mutations in PINK1 genes have provided a credible basis to a certain extent to meticulously monitor and comprehend the otherwise complex etiology of PD. PINK1 mutations were found to be severely damaging in C2-ceramide (neurotoxin) challenged brains thereby suggesting on the neuroprotective role of PINK1 in preventing mitochondrial dysfunction and reinforcing the anti- apoptotic and neuronal survival pathways such as Bcl-2 and PI3K/AKT (64). PINK1 and PARKIN are responsible for mitochondrial damage limitation during the active durations of stress and cooperate together in autophagy following mitochondrial injury. Examination of primary mouse cells acquired from PINK1- knockout mice directed that PARKIN induction and lysosomal translocation proceeded autonomous of PINK1. Moreover, suppression of the PI3K/AKT-mTOR pathway by therapeutic proxies can vary PARKIN expression accordingly. These results altogether validate that PARKIN and PINK1 are co-regulated during starvation and suggest a likely role of PI3K/AKT-mTOR in response to trophic signals and starvation stress (65).

**Fig. 2:**
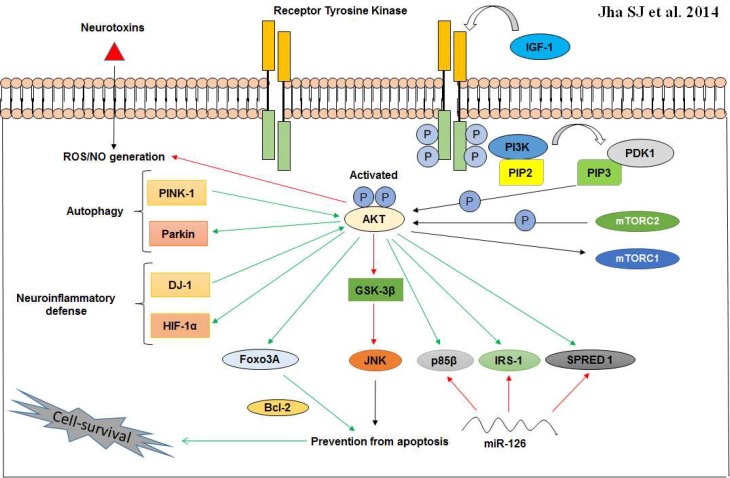
Neuroprotective cross-talk involving the cytoprotective PI3K/AKT pathway. AKT when optimally activated by phosphorylation at serine and threonine residues, can interact with a spectrum of molecules to erect an anti- inflammatory (DJ-1 and HIF-1α) and anti- apoptotic (Bcl-2) ambience in vulnerable neurons. In addition, phosphorylated AKT can also promote autophagy via PINK-1 and Parkin. IRS-1 activation takes place via IGF-1/AKT cascade and other AKT targets including p85β and SPRED 1 are known to be downregulated by miR-126 in PD neurons. Activation (green arrows); prevention or suppression (red arrows

PI3K/AKT pathway can also play a key role in IGF-mediated cell survival and prevention of apoptosis in MPP+ induced human neuroblastoma SH-EP1 cells. This defensive activity of AKT is principally reliant on the BIO mediated inactivation of GSK-3β, the result of which could imitate the protective influence of IGF-1 in SH-EP1 cells. Interestingly, the IGF-1 potentiated PI3K/AKT activity was found to further down regulate the JNK related apoptotic activity and this negative regulation was reported to be facilitated via AKT-dependent GSK-3β inactivation (Figure 2). Moreover, these results acknowledge that IGF-1 protects SH-EP1 cells from MPP+-induced apoptotic cell mortality via the cytoprotective PI3K/AKT/GSK-3β pathway involving GSK-3β inactivation (66-68). Most recently, it hes been studied in DA neuronal cell systems that upregulation of miR-126 impaired IGF-1 signalling and increased the susceptibility of such systems to 6-OHDA, possibly by stamping down factors involved in IGF-1/PI3K signalling, including its downstream targets p85β, IRS-1, and SPRED1. MicroRNAs (miRs/miRNAs) act as posttranscrip-tional regulators of gene expression and therefore, it is unsurprising that they could be critically modulating pathogenesis in PD. Notably, blocking miR-126 activity increased IGF-1 trophism and thereby combating the cataclysmic events of 6- OHDA. This result strongly ascertain the criticality IGF-1/ PI3K cascade in DA neuron maintenance and also suggests that the higher expression patterns of miR-126 may contribute towards DA neurodegeneration aided by downregulation of IGF-1/PI3K/AKT signalling (69).

Glial cell line-derived neurotrophic factor (GDNF) is necessary for DA neuronal maintenance and development. GDNF is normally found to be neuroprotective in animal models of PD, where selective DA neurodegeneration is a characteristic feature (70). GDNF can have potent neuroprotective effects in nigrostriatal DA neurons that are degraded in PD. H_2_O_2_ or l-3, 4-dihydroxyphenylalanine (l-DOPA) when used to injure DA neurons, prompts the release of soluble factors that signal the ventral midbrain astrocytes to upregulate GDNF concentration. Notably, PI3K pathway is central towards this mechanism of striatal GDNF up-regulation as triggered by H_2_O_2_. Conversely, diffusible factors released in the presence of l-DOPA- trigger GDNF expression via activation of the MAPK pathway (71). Another study attempted to decipher whether cadherin has a profound impact on PI3K/AKT activation in DA neurons mediated by the protective effects of GDNF. Cadherins are calcium-dependent adhesion proteins, and N-cadherins are expressed in DA neurons. Interestingly, the results of the investigation suggested that N-cadherin was indeed involved in PI3K/AKT activation in DA neurons triggered by GDNF (72). Aging mice which lacks DJ-1 and the GDNF-receptor Ret expression in the DA system exhibits loss of substantia nigra (SN) cell bodies, but not axons, compared to mice compromised only in Ret. This survival requirement for DJ-1 is specific for the most vulnerable GIRK2-positive populace in the SN which projects entirely to the striatum. Study on Drosophila genetics further revealed about the constitutively active Ret and associated Ras/ERK, but not PI3K/AKT, signalling, which interact genetically with DJ-1. A better understanding of the molecular connections between trophic signalling, cellular stress and aging could uncover new targets for drug development in PD (73).

## Neuroprotective molecules of relevance in p38 and PI3K/AKT mediated PD prognosis

Neurotherapeutics research in recent times have probed a spectrum of protective biomolecules which can either activate the PI3K/AKT cascade, while others assist in limiting the activation of p38 MAPK in neurons; Table 1 provides a list of neuro- protective therapeutic modalities that sum on either the misfiring p38 MAPK and/or PI3K/AKT cascades in some form or the other, en route to providing a resilient neuroprotec-tive shield against the hastened degeneration of neurons in PD. 

**Table 1:** Neuroprotective Biomolecules offering neuroprotection in neurotoxin challenged Parkinson’s model systems presumably via p38 MAPK and/or PI3K/AKT cascade

**Biomole-cules**	**Nature**	**PD Model** ** systems**	**Neurotoxin**	**Proteins involved**	**Mode of action**	**Refer-ences**
Guanine based purines	anti-apoptotic	SH-SY5Y	6-OHDA	AKT, p38, JNK, and Bcl-2	Triggers an early upsurge in the phosphorylation of Akt and subsequent activation of the cytoprotective PI3K/AKT/PKB pathway; prevents the 6-OHDA intermediated activation of p38 and JNK and cause an upsurge in the expression level of the anti-apoptotic Bcl-2 protein.	(9)
Human albumin	anti-oxidant and anti-apoptotic	PC12	6-OHDA	JNK, c-Jun, ERK, and p38 MAPK	Attenuates 6-OHDA-inflicted ROS generation and apoptosis; inhibits 6-OHDA-induced activation of JNK, c-Jun, ERK, and p38 MAPK signalling.	(74)
Peroxiredoxin (PRX-2)	anti-oxidant and anti-apoptotic	MN9D DA neurons	6-OHDA	ASK1, c-Jun , p38	Inhibits 6-OHDA-induced ASK1 activation by regulating the redox properties of the endogenous ASK1 inhibitor Trx; display significant anti-apoptotic properties via suppression of ASK1-dependent activation of the c-Jun N-terminal kinase/c-Jun and p38 pro-mortality cascades; lastly, PRX2 over expression preserves Trx in a reduced state by blocking the cysteine thiol-disulfide interchange, thus preventing the dissociation of thioredoxin from ASK1.	(75, 76)
NOSH-ASA (NO- and H2S-releasing hybrid of aspirin)	anti-inflamma-tory	IFNγ-stimulated human astroglia and U373 cells, SH-SY5Y		TNFα, IL-6 , P38 MAPK and NFκB	Results in reduced TNFα and IL-6 levels along with a concomitant deactivation of P38 MAPK and NFκB proteins.	(77)
Bu-7	anti-apototic	PC12	Rotenone	JNK,p38,p53,caspase-3,Bax,Bcl-2	Protects the cells from rotenone triggered apoptosis and subsequent death; limits the rotenone induced potential reduction in mitochondria of the treated cells, prevents the rotenone induced activation of JNK, p38, p53, cleaved caspase-3 and decreases the Bax/Bcl-2 ratio.	(78)
3,4-Dihydroxybenzalacetone (DBL)	anti-oxidant, anti-inflam-matory, and anti-tumori-genic	SH-SY5Y	6-OHDA	Akt, ERK, p38 MAPK, PI3K	Induce stress-associated kinases such as Akt, ERK, and p38 MAPK, and PI3K or Akt inhibitors, but not ERK, p38, or JNK inhibitors; activates the Nrf2/glutathione cascade via PI3K/Akt, and facilitates survival of SH-SY5Y cells.	(79)
Tetrahydroxystilbene glucoside (TSG)	anti-apoptotic	PC12 and mice	MPTP	DAT, AKT, GSK3β, Bcl-2, BAD, caspase-3 and caspae-9	Protects DA neurodegeneration by averting MPTP-induced reduction of SN tyrosine hydroxylase (TH)-positive cells and striatal dopaminergic transporter (DAT) protein expression; increase in striatal Akt and GSK3β phosphorylation, up-regulation of the Bcl-2/BAD ratio, and inhibition of caspase-9 and caspase-3 activity; offers neuroprotective effects against MPP-prompted damage and apoptosis in PC12 cells, presumably through PI3K/Akt. Activation.	(80)
Tyrosol [2-(4-ydroxy-phenyl) ethanol]	anti-apoptotic	CATH.a	MPP(+)	PI3K, AKT, SOD-1, SOD-2 and DJ-1	Is neuroprotective against (MPP(+))-induced CATH.a neuronal death in a dose dependant manner by its ability to activate the PI3K/Akt signalling cascade; Tyrosol also upregulate SOD-1, SOD-2 and DJ-1.	(81)
Oxicam non-stero-idal anti-inflamma-tory drugs (NSAIDs)	anti-inflammtory	SH-SY5Y and mice	MPTP	PI3K, AKT, and COX	Offers protection via the PI3K/Akt cascade independentally of cyclooxygenase (COX) inhibition.	(82)
Tocotrien-ols (T3s)	anti-oxidant	SH-SY5Y	MPP(+)	ER β and PI3K/AKT	γT3 and δT3 treatments triggers the PI3K/Akt signalling module and this could perhaps be under the con-trol of estrogen receptor (ER) β; ER β being an upstream regulator of PI3K/Akt; T3s and, especially, γT3/δT3 in conjunction with the ac-tivation of ERβ/PI3K/Akt cascade, display not only antioxidant activity but also offers a receptor signal-mediated neuroprotection.	(83)
Danshensu (beta-3,4-dihydroxyphenyl-lactic acid)	ROS scavenger and anti-oxidant	PC12 and Zebra fish DA neurons	6-OHDA	PI3K/AKT, Nrf-2, and HO-1	Induces Akt phosphorylation, and the induced cytoprotective effects are reversed by PI3K, Akt and HO-1 inhibitors; enhances HO-1 expression in order to suppress 6-OHDA-induced oxidative stress via PI3K/Akt/Nrf2 cascade.	(84)
Puerarin	anti-oxidant	Mice	MPTP	PI3K/AKT, GSH, and GDNF	Puerarin administration enhances glutathione (GSH) activity, glial cell line-derived neurotrophic factor (GDNF) expression and activates the PI3K/Akt pathway; dampens MPTP-reduced lysosome-associated membrane protein type 2A (Lamp 2A) expression.	(85)
Eucommia ulmoides Oliv. Bark. (EUE)	anti-oxidant	SH-SY5Y	6-OHDA	JNK, PI3K/Akt, GSK-3β, and NF-κB	EUE reduces 6-OHDA-induced ROS formation, mitochondrial dysfunction, cell death and cytotoxicity; mitigates oxidative stress through induction of JNK, PI3K/Akt, GSK-3β, and NF-κB cascades.	(86)
Rotigotine	anti-oxidant, anti-apototic	primary dopaminergic cultures	glutamate	D3 receptor, AKT, and GSK-3-β	The molecule most likely stimulates the dopamine D3 receptor; abates the production and accumulation of superoxide radicals; consistent exposure to Rotigotine promotes Akt phosporylation, and results in deactivation of the pro-apoptotic component GSK-3-β.	(87)
Squamos-amide derivative FLZ		Rats	6-OHDA	PI3K/AKT, α-Syn, and TH	FLZ protects TH activity and DA neurons by diminishing α-synuclein (α-Syn) expression and the cooperation between α-Syn and TH, FLZ neuroprotection involves the PI3K/AKT cascade and blocking the cascade attenuates α-Syn expression and subsequently the protection offered by FLZ is lost.	(67)

In conclusion environmental exposures to toxic mediators lead to neurodegenerative sickness that has common pathophysiology and clinical findings with PD. It is conjectured that decisive factors like microglia activation, neuroinflam-mation, oxidative stress due to ROS accumulation, NO activity, and neuronal apoptosis resulting from all these modes are at the base of DA neuronal toxicity and subsequent damage in Parkinson’s brains. However, the precise identity and functional prototypes of molecular intermediates leading to neuronal mortality still remains to be deciphered. Recent studies have highlighted the fundamental role of p38 MAPK in controlling all of the above detrimental consequences, and thereby in the process upset DA neuronal homeostasis, which ultimately progresses to an advanced diseased state and incurable neurodegeneration. Using cell systems like SH-SY5Y and PC12, several factors like inducible NO synthase, ATF6α (oxidative stress), IL-1β, and TNF-α (neuro-inflammation), and Bax and ASK1 (apoptosis) were found to be relevant in DA neuronal death. Unprecedented observations that these factors operate in unison with the p38 MAPK cascade strongly advocate the cyto-destructive nature of the cascade in degenerating neurons. Conversely, the normal functioning of the PI3K/AKT pathway ensures that the neuroprotective defence is active in order to negate the destructive aftermath of p38 MAPK activation in degenerating neurons. In the process, the activated AKT interacts with several mediators like JNK, FoxO, GSK3β, etc., thereby to render neuroprotection by limiting apoptosis, preventing microglia activation and neuroinflammation, preventing ROS accumulation and by keeping oxidative stress levels under check. However, the pathway is misregulated in PD brains and eventually it fails to render its protective veneer in traumatized brains. It is thereby necessary to identify modalities which can repair the misbalanced p38/PI3K interactome in order to limit poor prognosis in PD patients.
